# EGFR Mutation and TKI Treatment Promote Secretion of Small Extracellular Vesicle PD-L1 and Contribute to Immunosuppression in NSCLC

**DOI:** 10.3390/biom14070820

**Published:** 2024-07-09

**Authors:** Hai-Ming Liu, Zi-Li Yu, Hou-Fu Xia, Lin-Zhou Zhang, Qiu-Yun Fu, Yi Wang, Hong-Yun Gong, Gang Chen

**Affiliations:** 1 State Key Laboratory of Oral & Maxillofacial Reconstruction and Regeneration, Key Laboratory of Oral Biomedicine Ministry of Education, Hubei Key Laboratory of Stomatology, School and Hospital of Stomatology, Wuhan University, Wuhan 430079, China; 2 Department of Oral and Maxillofacial Surgery, School and Hospital of Stomatology, Wuhan University, Wuhan 430079, China; 3 Cancer Center, Renmin Hospital of Wuhan University, Wuhan 430060, China; 4 TaiKang Center for Life and Medical Sciences, Wuhan University, Wuhan 430071, China; 5 Frontier Science Center for Immunology and Metabolism, Wuhan University, Wuhan 430071, China

**Keywords:** *EGFR*-mutant NSCLC, TKIs, sEV PD-L1, immunotherapy

## Abstract

In Asian populations with non-small-cell lung cancer (NSCLC), *EGFR* mutations are highly prevalent, occurring in roughly half of these patients. Studies have revealed that individuals with *EGFR* mutation typically fare worse with immunotherapy. In patients who received EGFR tyrosine kinase inhibitor (TKI) treatment followed by anti-PD-1 therapy, poor results were observed. The underlying mechanism remains unclear. We used high-resolution flow cytometry and ELISA to detect the circulating level of small extracellular vesicle (sEV) PD-L1 in NSCLC individuals with *EGFR* mutations before and after receiving TKIs. The secretion amount of sEV PD-L1 of lung cancer cell lines with *EGFR* mutations under TKI treatment or not were detected using high-resolution flow cytometry and Western blotting. The results revealed that patients harboring *EGFR* mutations exhibit increased levels of sEV PD-L1 in circulation, which inversely correlated with the presence of CD8^+^ T cells in tumor tissues. Furthermore, tumor cells carrying *EGFR* mutations secrete a higher quantity of PD-L1-positive sEVs. TKI treatment appeared to amplify the levels of PD-L1-positive sEVs in the bloodstream. Mutation-induced and TKI-induced sEVs substantially impaired the functionality of CD8^+^ T cells. Importantly, our findings indicated that *EGFR* mutations and TKI therapies promote secretion of PD-L1-positive sEVs via distinct molecular mechanisms, namely the HRS and ALIX pathways, respectively. In conclusion, the increased secretion of PD-L1-positive sEVs, prompted by genetic alterations and TKI administration, may contribute to the limited efficacy of immunotherapy observed in *EGFR*-mutant patients and patients who have received TKI treatment.

## 1. Introduction

Non-small-cell lung cancer (NSCLC) accounts for about 85% of all primary lung cancers, making it one of the deadliest cancers globally [1]. In Asian populations with NSCLC, mutations in the Epidermal Growth Factor Receptor (*EGFR*) are highly prevalent, occurring in roughly half of these patients [2]. EGFR, a transmembrane protein part of the ERBB receptor tyrosine kinase family, becomes continuously activated by kinase domain mutations. This activation is critical for NSCLC cellular survival, whereas blocking EGFR activity can deeply suppress tumor proliferation. Significant extended survival was observed in patients with advanced NSCLC harboring *EGFR* mutations treated with EGFR tyrosine kinase inhibitors (TKIs), establishing these inhibitors as the frontline treatment for patients with compatible mutations [3]. First-generation EGFR inhibitors like gefitinib and erlotinib, as well as second- and third-generation products such as afatinib and osimertinib, have all demonstrated notable clinical efficacy. Unfortunately, resistance to these agents often develops over time, even in those who initially respond well, leaving few options after targeted therapy failure [4]. As a consequence, the search for new treatment strategies to improve outcomes for these patients is critical [5].

Immunotherapy, especially with the anti-PD-1/PD-L1 inhibitors, has revolutionized the therapeutic landscape for various cancers, including NSCLC. These treatments unleash the body’s immune response to recognize and eradicate cancer cells. For mutant *EGFR*, preclinical studies indicate its potential to enhance PD-L1 expression within tumors via pathways such as PI3K-AKT mTOR, Hippo YAP, and IL6-JAK-STAT3, thus providing a theoretical basis for considering PD-1/PD-L1 inhibitors in *EGFR*-mutant NSCLC [6,7]. Nevertheless, clinical observations have indicated that patients with *EGFR* mutations generally exhibit poorer responses to immunotherapy. This trend is illustrated in the Checkmate-012 trial, where individuals with *EGFR* mutations demonstrated weaker responses compared to those with wild-type (WT) *EGFR* [8]. Moreover, even when PD-L1 expression is high, those harboring *EGFR* mutations may still respond less favorably to immunotherapy compared to the general cancer patients. This probably results from *EGFR*-mutant cancers’ tendency to foster an immunosuppressive tumor microenvironment, a potential cause of the diminished immunotherapy response [9]. The impact of *EGFR* mutations on the response to PD-1/PD-L1 inhibitors remains a subject of ongoing research.

TKI treatment might reverse the immunosuppressive milieu associated with *EGFR* mutations. Research suggests that TKIs can positively alter the tumor immune environment, influencing tumor antigen presentation and the infiltration of immune cells. Early in the course of TKI treatment, there appears to be a beneficial but transient shift in the immune environment: increased populations of cytotoxic CD8^+^ T cells, greater activity in dendritic cells, reduction of Foxp3^+^ Tregs, and reduced M2-like macrophage polarization [10]. These observations imply that TKIs could sensitize *EGFR*-mutant patients to PD-1/PD-L1 inhibitors. However, findings from a KEYNOTE-001 clinical trial painted a discouraging picture: post-TKI treatment and subsequent pembrolizumab administration yielded poor results, with progression and survival times far below expectations [11]. This outcome may be due to the absence of sustained positive changes in the immune effector cell population during prolonged TKI therapy, alongside a reduction in CD8^+^ T cell and macrophage infiltration and an increase in tumor cell PD-L1 expression upon developing TKI resistance [12,13]. Nevertheless, the precise mechanisms behind this remain elusive, posing an ongoing challenge in the treatment of these patients. These findings also prompt the question of why patients with mutant *EGFR* and those who have been treated with TKIs both exhibit poor responses to immunotherapy.

Small extracellular vesicles (sEVs), omnipresent in biological fluids like blood, saliva, and urine, are membrane-bound vesicles released by cells. They serve as essential mediators of intercellular communication, ferrying a multitude of bioactive compounds and participating in various physiological and pathological processes [14]. Our recent findings suggest that cancer cells release PD-L1-positive sEVs into the bloodstream, potentially serving as a systematic mechanism to counteract the anti-tumor immune response [15]. PD-L1-positive sEVs may contribute to the immunotherapy resistance in patients with *EGFR* mutations, which aroused our interest.

## 2. Materials and Methods

### 2.1. Patients and Specimen Collection

Peripheral blood specimens and pathological slides of the NSCLC patients were collected in the Renmin Hospital, Wuhan University. Ethical approvals were granted by the Ethics Committee of Renmin Hospital (WDRY2021-K021). This study was done in accordance with the Declaration of Helsinki for the utilization of the patient samples. NSCLC patients diagnosed as *EGFR*-mutant received oral gefitinib 250 mg daily for three months. Tumor biopsies and peripheral blood were collected at the diagnostic stage and the end-of-treatment phase. Tumor samples and peripheral blood of *EGFR*-WT NSCLC patients were collected at the time of surgery. Pathological slides were collected from the department of pathology.

### 2.2. Isolation of Extracellular Vesicles

Purification of small extracellular vesicles was performed as previously described [16]. Detailed steps are provided in the Supplementary Materials.

### 2.3. Nanoparticle Tracking Analysis (NTA)

Small extracellular vesicles were detected using Particle Metrix, Inning am Ammersee, Germany (ZetaView). The number and size of the small extracellular vesicles were revealed by the results.

### 2.4. Enzyme-Linked Immunosorbent Assay (ELISA)

The plasma samples were utilized to collect the sEVs. Detailed steps of ELISA are provided in the Supplementary Materials. To quantify the results, the plate was measured using a microplate reader (BioTek, Winooski, VT, USA).

### 2.5. Cell Culture and Reagents

Both H1264 and H1975 NSCLC cell lines were purchased from the American Type Culture Collection (ATCC). For construction of *HRS* and *ALIX* knockdown H1975 cell lines, cells were infected with lentivirus and selected with puromycin. For construction of *EGFR*-mutant H1264 cell lines, cells were infected with lentivirus and selected with blasticidin.

### 2.6. Immunohistochemistry (IHC)

Detailed steps and primary antibodies are provided in the Supplementary Materials. IHC staining was performed as previously mentioned [17].

### 2.7. Western Blotting Analysis

The lysis buffer was used to extract the total protein. The BCA assay was used to quantify the protein content. Detailed steps are provided in the Supplementary Materials. Finally, the ECL detection system was used to detect the immunoreactive bands and the emitted light was recorded on film. Original western blots can be found at Supplementary Materials.

### 2.8. Coculture System

Peripheral blood mononuclear cells (PBMCs) were purified from the peripheral blood of healthy donors. PBMCs were activated using CD3/CD28 mAb. Then PBMCs were treated with 50 μg sEVs and cocultured for 48 h. 

### 2.9. Flow Cytometry Analysis

Cells were harvested and permeabilized for staining. The staining was performed using anti-human antibodies: Granzyme B (BioLegend, San Diego, CA, USA), Ki67 (BioLegend), and CD8 (BioLegend). Results were acquired using flow cytometer (Beckman, Brea, CA, USA). 

The purified sEVs from plasma samples were stained with anti-human antibody: PD-L1 (BioLegend, San Diego, CA, USA). And then the samples were analyzed using A60Micro-PLUS (Apogee, Santa Monica, CA, USA). Finally, the data were acquired and analyzed using FlowJo software 10.0 (BD, East Rutherford, NJ, USA).

### 2.10. Statistical Analysis

Student’s *t* test and one-way ANOVA were used for statistical comparisons. Spearman’s rank correlation test was used for correlation analysis. Statistical significance was defined as *p* < 0.05. Data were analyzed using GraphPad Prism software 10.0 (Inc., La Jolla, CA, USA).

## 3. Results

### 3.1. Increased Circulating sEV PD-L1 Levels in Patients with EGFR Mutations

**Figure 1:**
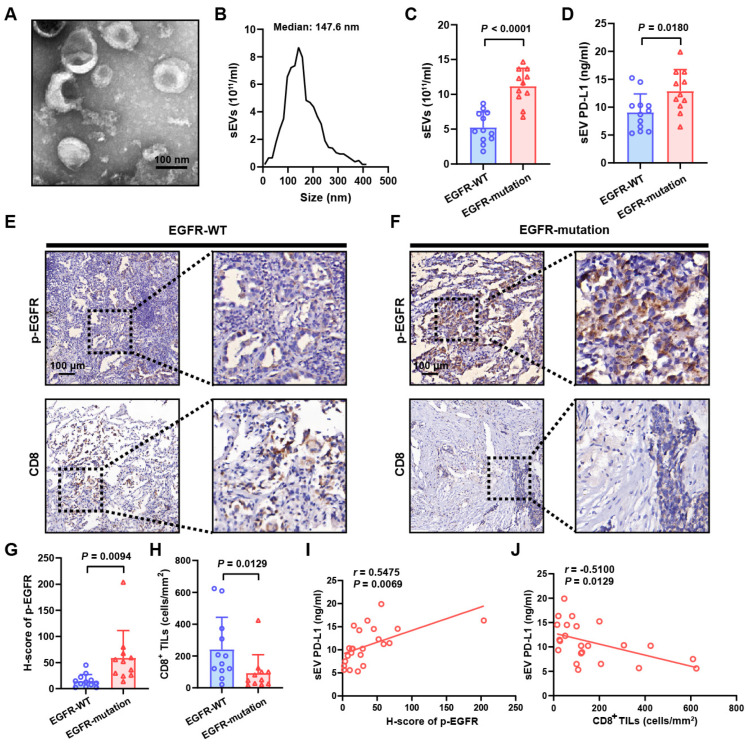
Elevated levels of circulating sEV PD-L1 detected in patients with *EGFR* mutations. (**A**) The representative image of purified circulating sEVs of NSCLC patients analyzed by TEM. The scale bar represents 100 nm. (**B**) The size and particle number of circulating sEVs of NSCLC patients. (**C**) Comparison of the concentration of circulating sEVs between NSCLC patients harboring *EGFR*-WT and *EGFR* mutations. (**D**) ELISA assay-based analysis of the PD-L1 expression on circulating sEVs of patients harboring *EGFR*-WT and *EGFR*-mutant NSCLC. (**E**) Representative immunohistochemical staining for p-EGFR and CD8 tumor-infiltrating lymphocytes (TILs) in *EGFR*-WT tissue. The scale bar represents 100 μm. (**F**) Representative immunohistochemical staining for p-EGFR and CD8 in *EGFR*-mutant tissue. The scale bar represents 100 μm. (**G**) Quantitative analysis of p-EGFR expression in *EGFR*-WT and *EGFR*-mutant tumor tissues. (**H**) Quantification of CD8^+^ TILs in *EGFR*-WT and *EGFR*-mutant tumor tissues. (**I**) The correlation between the level of circulating sEV PD-L1 and p-EGFR expression in tumor tissues. (**J**) The correlation between the level of circulating sEV PD-L1 and the number of CD8^+^ TILs in tumor tissues.

To illuminate the connection between sEV PD-L1 secretion and *EGFR* mutations, we isolated sEVs from the plasma samples of NSCLC patients who either had the wild-type *EGFR* (*EGFR*-WT) or mutated *EGFR* (*EGFR*-mutation, L858R mutation). These sEVs were purified by differential centrifugation and validated by transmission electron microscopy and nanoparticle tracking analysis (Figure 1A,B). A higher number of circulating sEVs was detected in patients with *EGFR* mutations compared to those with *EGFR*-WT (Figure 1C). Circulating sEV PD-L1 levels were measured by ELISA tests, revealing a significant elevation in the *EGFR*-mutant cohort (Figure 1D). Clinical tumor specimens were subjected to immunohistochemical analysis, enabling the assessment of phospho-EGFR (p-EGFR) expression levels and the infiltration level of CD8^+^ tumor-infiltrating lymphocytes (TILs). It was observed that patients harboring *EGFR* mutations exhibited higher levels of p-EGFR within tumor tissues as opposed to those with the wild-type allele (Figure 1E–G). Furthermore, a decreased infiltration of CD8^+^ TILs was noted in the *EGFR*-mutant NSCLC patients (Figure 1E,F,H). A positive correlation was found between the levels of circulating sEV PD-L1 and p-EGFR expression (Figure 1I). Conversely, an inverse relationship was discovered between the levels of circulating sEV PD-L1 and the infiltration of CD8^+^ TILs (Figure 1J). These results have suggested potential connections between *EGFR* mutations and circulating sEV PD-L1, which have been shown to have a negative regulatory effect on anti-tumor immunity.

### 3.2. Mutation of EGFR Promoted Secretion of sEV PD-L1 via HRS

**Figure 2:**
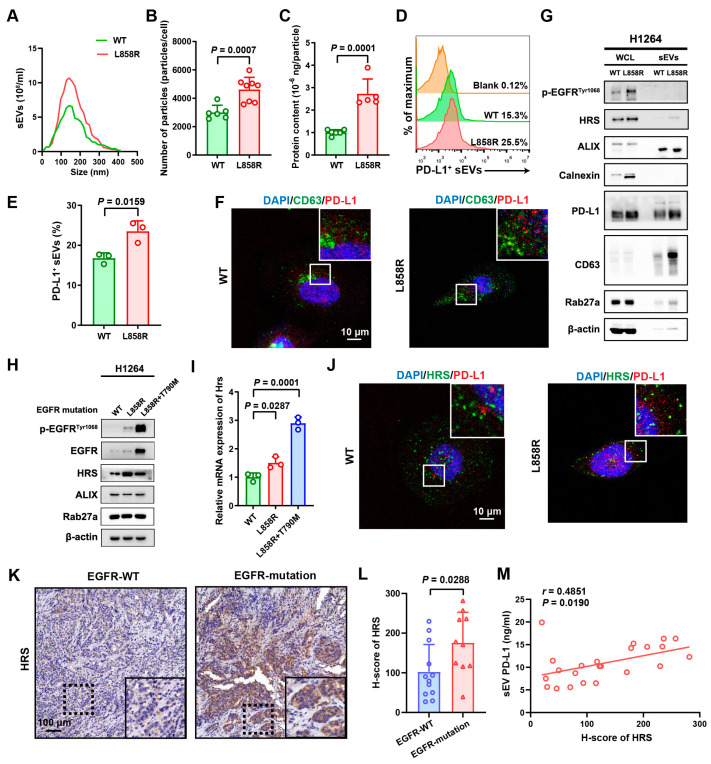
*EGFR* mutation enhanced secretion of sEV PD-L1. (**A**) Size distribution and particle number of purified sEVs from WT and L858R mutant H1264 cell lines, as analyzed by NTA. (**B**) Number of particles detected in the culture supernatant from the WT and L858R mutant H1264 cell lines. (**C**) Protein content of particles detected in the culture supernatant from WT and L858R mutant H1264 cell lines. (**D**) Representative histograms from high-resolution flow cytometry of PD-L1-positive sEVs in the supernatants from WT and L858R mutant H1264 cell lines. (**E**) Comparison of the percentage of PD-L1-positive sEVs between WT and L858R mutant H1264 cell lines. (**F**) Immunofluorescence staining for cellular PD-L1 and CD63 in WT and L858R mutant H1264 cell lines. (**G**) The protein expression level of HRS and PD-L1 in WT and L858R mutant H1264 cell lines and derived sEVs. (**H**) The protein expression level of proteins HRS, ALIX, and Rab27a in WT, L858R, and L858R+T790M mutant H1264 cell lines. (**I**) RT-PCR for HRS in WT, L858R, and L858R+T790M mutant H1264 cell lines. (**J**) Immunofluorescence staining for cellular PD-L1 and HRS in WT and L858R mutant H1264 cell lines. (**K**) Immunohistochemical staining of HRS in *EGFR*-WT and *EGFR*-mutant tumor tissues. (**L**) Quantitative analysis of HRS expression in *EGFR*-WT and *EGFR*-mutant tumor tissues. (**M**) The correlation between the expression of HRS in tumor tissues and the level of circulating sEV PD-L1.

The heightened levels of sEV PD-L1 in patients with *EGFR* mutations led us to hypothesize that *EGFR* mutations might enhance the release of PD-L1-positive sEVs. To investigate this theory, we developed the human NSCLC H1264 cell line carrying the L858R mutation, which is the major type of common *EGFR* mutation. As expected, we observed that PD-L1 levels in NSCLC H1264 cells were considerably upregulated by the L858R mutation, as supported by PCR assays (Figure S1A), and flow cytometry analysis (Figure S1B,C). Subsequently, we harvested sEVs from the conditioned medium of the H1264 cells and assessed the particle count and total protein concentration using the BCA assay and NTA, respectively. Our data indicated that the L858R mutation considerably amplified the secretion of total sEVs by H1264 cells (Figure 2A–C). Additionally, we employed high-resolution flow cytometry to gauge the proportion of PD-L1-positive sEVs within the total sEV population and observed an increase from 15.3% (WT control) to 25.5% due to the L858R mutation (Figure 2D,E). Confocal microscopy also highlighted increased co-localization of CD63 and PD-L1 in H1264 cells with L858R mutation (Figure 2F). Western blotting analysis further validated that both PD-L1 and CD63 (a hallmark marker of sEVs) were markedly elevated in sEVs from H1264 cells with the L858R mutation when compared to the control (Figure 2G), reinforcing the conclusion that *EGFR* mutations expedite the release of sEV PD-L1. 

*EGFR* mutations are widely recognized to raise phosphorylation levels of the receptor, which in consequence provokes shifts in downstream signaling cascades. Pursuing this line of inquiry, we examined whether epidermal growth factor (EGF), the natural ligand of EGFR that induces receptor phosphorylation upon binding, could similarly stimulate the release of PD-L1-positive sEVs. As anticipated, EGF stimulation significantly promoted the phosphorylation of EGFR in H1264 cells (Figure S2A). Furthermore, we noted a substantial elevation in PD-L1 expression in H1264 cells following EGF treatment, which was corroborated by Western blotting analysis (Figure S2A), PCR assays (Figure S2B), and flow cytometry (Figure S2C,D). Cumulative evidence from BCA assays, NTA, Western blotting, and high-resolution flow cytometry collectively confirmed that EGF treatment further heightened the secretion of sEV PD-L1 (Figure S3A–E). The collective findings clearly illustrate that phosphorylation, elicited by either *EGFR* mutations or engagement with its ligand EGF, facilitates the enhanced secretion level of sEV PD-L1 in NSCLC cell lines.

Drawing from the literature, proteins HRS, ALIX, and RAB27a have been implicated in the regulation of PD-L1-positive sEV release. Accordingly, we explored the contributions of these pivotal molecules in the release of sEV PD-L1 mediated by EGFR phosphorylation. The outcomes from the Western blotting analysis indicated a marked elevation in HRS levels upon either *EGFR* mutation or EGF stimulation in NSCLC cells, whereas levels of RAB27a and ALIX remained unchanged (Figure 2H and Figure S4A–D). Accompanied by the EGFR phosphorylation, both L858R and L858R+T790M mutation exhibited a greater increase in HRS levels when compared to the *EGFR*-WT. These findings were echoed by PCR results for mRNA levels (Figure 2I and Figure S4E,F). Immunofluorescence staining highlighted heightened co-localization of HRS with PD-L1 in the presence of the L858R mutation (Figure 2J). We further assessed the link between sEV PD-L1 levels and expression of HRS in tumor tissue via immunohistochemistry staining. IHC analysis of *EGFR*-mutant specimens revealed markedly increased HRS expression as opposed to *EGFR*-WT tissues (Figure 2K,L). Notably, levels of circulating sEV PD-L1 were found to positively correlate with HRS presence in tumor samples (Figure 2M). These cumulative findings point to a significant role for HRS in the EGFR phosphorylation-mediated release of PD-L1-positive sEVs.

### 3.3. TKI Treatment Promoted Secretion of sEV PD-L1 via ALIX

**Figure 3:**
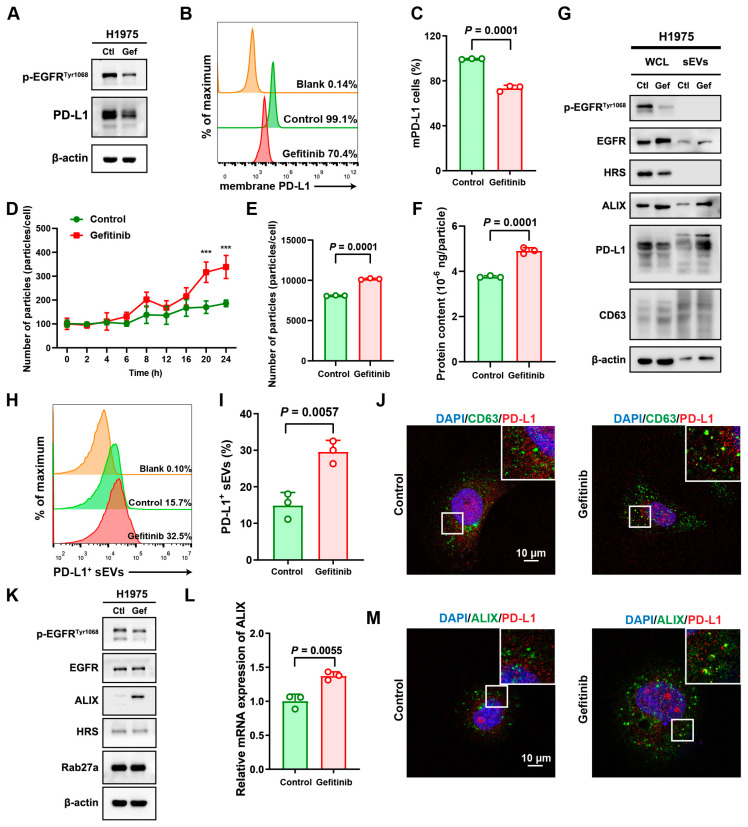
TKI treatment promoted secretion of sEV PD-L1 via ALIX. (**A**) The protein expression level of PD-L1 in H1975 cell line treated with gefitinib. (**B**) Representative histograms showing the membrane PD-L1 of H1975 cell line treated with gefitinib analyzed by flow cytometry. (**C**) Quantitative analysis of the percentage of membrane PD-L1-positive H1975 cell line treated with gefitinib. (**D**) Dynamically monitoring the particles in the supernatant of cell line H1975 under gefitinib treatment by NTA. *** indicates *p* < 0.001 (**E**) Number of particles detected in the cultural supernatant of H1975 cell line treated with gefitinib. (**F**) Protein content of particles detected in the cultural supernatant of H1975 cell line treated with gefitinib. (**G**) The protein expression level of ALIX and PD-L1 in H1975 cell line and the derived sEVs under gefitinib treatment. (**H**) Representative histograms of high-resolution flow cytometry for the proportion of PD-L1-positive sEVs from the supernatant of H1975 cell line treated with gefitinib. (**I**) Quantitative analysis of the percentage of PD-L1-positive sEVs. (**J**) Immunofluorescence staining of cellular PD-L1 and CD63 in H1975 cell line treated with gefitinib. (**K**) Western blotting analysis of proteins HRS, ALIX, and Rab27a in H1975 cell line treated with gefitinib. (**L**) RT-PCR of ALIX in H1975 cell line treated with gefitinib. (**M**) Immunofluorescence staining of cellular PD-L1 and ALIX in H1975 cell line treated with gefitinib.

The findings that EGFR phosphorylation, triggered by either *EGFR* mutations or binding with its ligand EGF, enhanced the release of sEV PD-L1 led to the hypothesis that inhibiting EGFR via TKI therapy could potentially curb sEV PD-L1 secretion. Addressing this hypothesis, we treated H1975 cells with gefitinib. As an *EGFR*-L858R+T790M mutation NSCLC cell line known for its TKI resistance, gefitinib did not significantly impact cell viability or clonogenicity (Figure S5A,B). Western blotting analysis revealed a clear reduction in H1975 cells’ EGFR phosphorylation after gefitinib exposure (Figure 3A).

Subsequently, we investigated cellular PD-L1 levels in H1975 cells post-gefitinib-treatment and found a significant downregulation, evidenced by Western blotting (Figure 3A). This observation was further substantiated by flow cytometry, which indicated a decrease in the proportion of PD-L1-positive cells following gefitinib exposure (Figure 3B,C). Additionally, over a 24 h period, we dynamically monitored the supernatant of the H1975 cell culture and noticed a marked increase in particle count with gefitinib treatment in comparison to the control (Figure 3D). This effect persisted even when normalizing sEV numbers to cell count (Figure 3E). Moreover, the protein content within the sEVs was also found to be higher upon gefitinib treatment (Figure 3F), suggesting that while cellular PD-L1 levels decreased, TKI treatment stimulated the secretion of sEVs. The PD-L1 levels within the sEVs were indeed significantly higher post-treatment according to Western blotting analysis (Figure 3G), with flow cytometry revealing an increase of PD-L1-positive sEVs from 15.7% in controls to 32.5% following gefitinib treatment (Figure 3H,I). Immunofluorescence staining emphasized more pronounced co-localization of CD63 with PD-L1 in the gefitinib context, indicating that PD-L1 was being secreted via sEVs in H1975 cells (Figure 3J). 

Then, we identified which pivotal molecules in the release of sEV PD-L1 were mediated by TKI treatment. Both Western blotting and RT-PCR analyses showed a significant upsurge in ALIX expression upon gefitinib treatment in H1975 cells, while levels of RAB27a and HRS were unaffected (Figure 3K,L and Figure S6A,B). A parallel was noted in H1975 cells treated with another TKI, afatinib (Figure S6C–E). Immunofluorescence staining presented increased ALIX and PD-L1 co-localization under gefitinib treatment (Figure 3M). In summary, the collected data suggest that TKI treatment encourages the release of sEV PD-L1 rather than suppressing it, with ALIX likely playing a part in this process.

### 3.4. TKI Treatment Increased the Level of Circulating sEV PD-L1 and Tissue ALIX in NSCLC Patients

**Figure 4:**
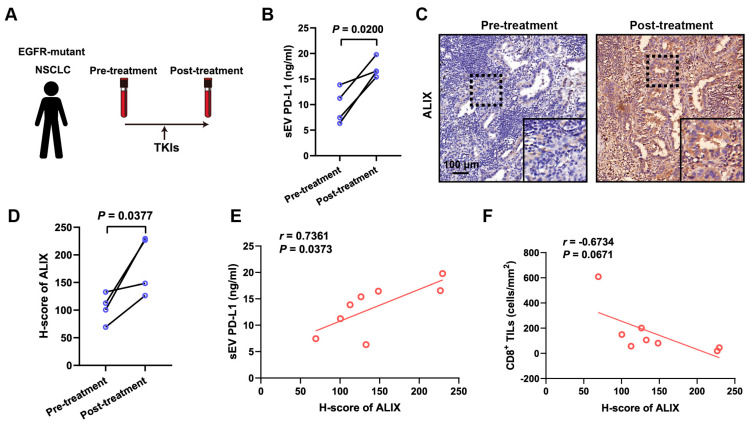
The level of circulating sEV PD-L1 and expression of ALIX were elevated in NSCLC patients following TKI treatment. (**A**) Sampling diagram for the treatment and sample collection of NSCLC patients. (**B**) ELISA-based quantification of sEV PD-L1 pre-treatment and post-treatment. (**C**) Immunohistochemical staining of ALIX in tumor tissues from NSCLC patients pre- and post-TKI-therapy. (**D**) Quantification of ALIX expression by IHC in pre-treatment and post-treatment phases. (**E**) The correlation between the level of circulating sEV PD-L1 and the expression of ALIX. (**F**) The correlation between CD8^+^ tumor-infiltrating lymphocyte density and ALIX expression in tumor tissues.

To further validate our findings in NSCLC patients, we procured paired peripheral blood and tissue samples before and after TKI therapy (Figure 4A). We then isolated sEVs from the plasma samples of NSCLC patients, both pre-treatment and after treatment, using differential centrifugation and quantified circulating sEV PD-L1 levels. Our results showed that the level of circulating sEV PD-L1 significantly increased following TKI therapy (Figure 4B). Furthermore, to investigate the relationship between ALIX expression and circulating sEV PD-L1 levels at the tissue level, we performed IHC staining of ALIX in tumor tissues of NSCLC patients. We observed a significant upregulation of ALIX expression in paired tumor tissues of NSCLC patients post-TKI therapy (Figure 4C,D). Additionally, there was a positive correlation between ALIX expression in tissues and the levels of circulating sEV PD-L1 (Figure 4E). In line with previous discussions, higher levels of circulating PD-L1-positive sEVs contributed to an immunosuppressive microenvironment, and we found that ALIX expression showed an inverse correlation with CD8^+^ TILs presence (Figure 4F). These results demonstrated that TKI treatment increased the level of circulating PD-L1-positive sEVs in NSCLC patients.

### 3.5. Knockdown of HRS and ALIX Inhibited the Secretion of sEV PD-L1 under Activation or Inhibition of EGFR Activity, Respectively

**Figure 5:**
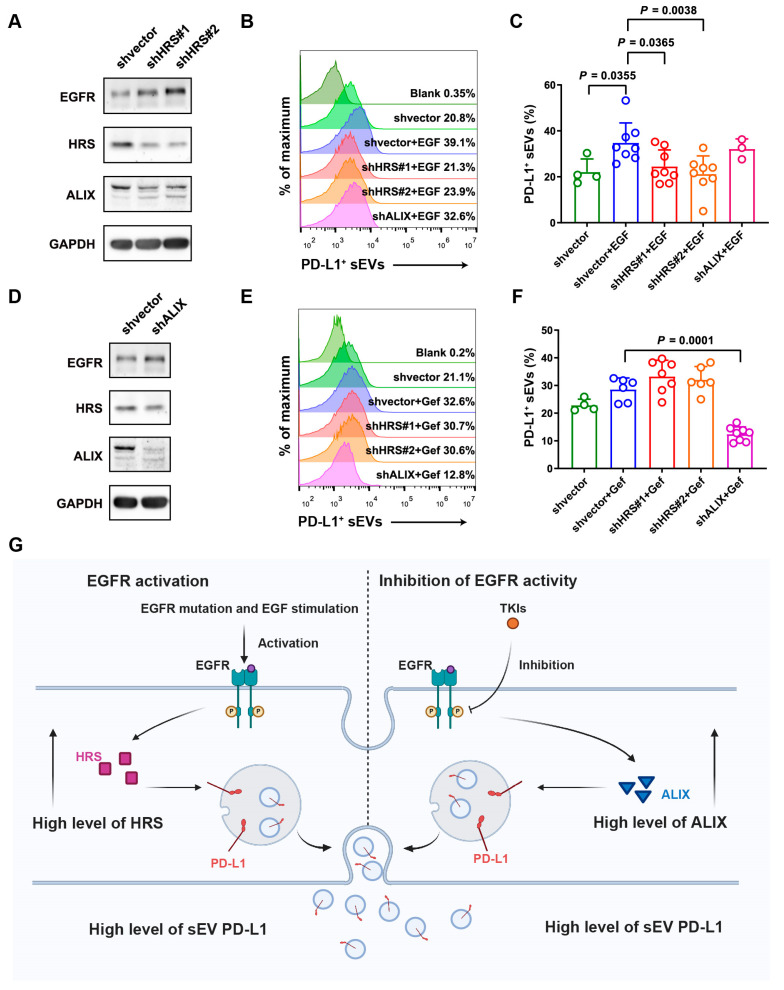
Knockdown of *HRS* and *ALIX* inhibited the secretion of sEV PD-L1 under activation or inhibition of EGFR activity, respectively. (**A**) The protein expression level of HRS and ALIX in the *HRS* knockdown cell lines. (**B**) High-resolution flow cytometry analysis of sEV PD-L1 in *HRS* and *ALIX* knockdown cell lines stimulated with EGF. (**C**) Quantification of the percentage of PD-L1-positive sEVs in the culture supernatant of cell lines treated with EGF. (**D**) The protein expression level of HRS and ALIX in the *ALIX* knockdown cell lines. (**E**) High-resolution flow cytometry analysis of sEV PD-L1 in *HRS* and *ALIX* knockdown cell lines treated with gefitinib. (**F**) Quantitative analysis of the percentage of PD-L1-positive sEVs in the culture supernatant of cell lines treated with gefitinib. (**G**) Illustration of the underlying mechanism of the secretion of sEV PD-L1 under activation and inhibition of EGFR.

To further investigate the roles of HRS and ALIX in the increased secretion of sEV PD-L1, associated with both the activation and inhibition of EGFR activity, we established *HRS* and *ALIX* knockdown NSCLC cell lines (Figure 5A,D). We then explored the secretion level of sEV PD-L1 in these knockdown cells under conditions of EGFR activation or inhibition. sEVs were collected from the supernatants of *HRS* and *ALIX* knockdown cell lines treated with EGF and gefitinib. The percentage of PD-L1-positive sEVs in the culture supernatant was determined using high-resolution flow cytometry. The results demonstrated that the knockdown of *HRS* significantly inhibited the secretion of PD-L1-positive sEVs upon EGF stimulation, whereas *ALIX* knockdown had a negligible effect on their secretion (Figure 5B,C). In contrast, further analysis indicated that the knockdown of *ALIX*, but not *HRS*, significantly restrained the TKI-induced secretion of PD-L1-positive sEVs (Figure 5E,F). These findings provided additional evidence that EGFR activation enhances the secretion level of sEV PD-L1 by modulating HRS expression. Conversely, the inhibition of EGFR signaling boosted the release of PD-L1-positive sEVs through the modulation of ALIX expression. Intriguingly, both the activation and inhibition of EGFR signaling appeared to stimulate the secretion of PD-L1-positive sEVs (Figure 5G).

### 3.6. Both EGFR Mutation and TKI Treatment Exacerbated Immunosuppressive Effects of sEV PD-L1

**Figure 6:**
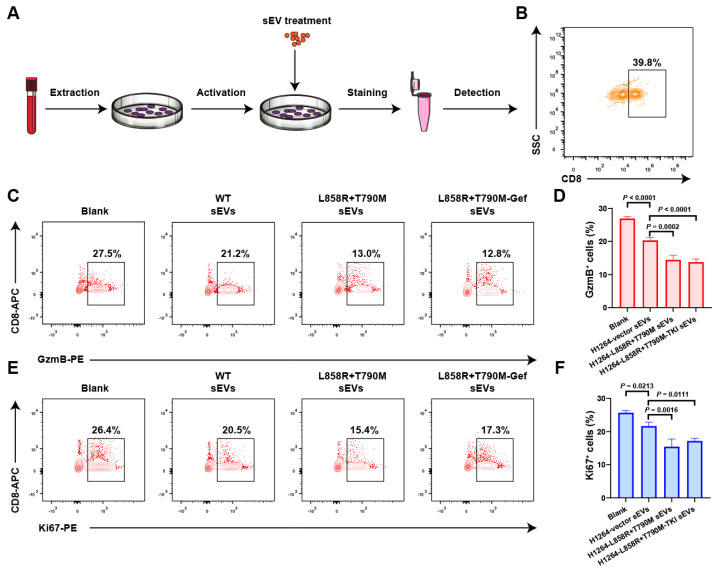
Both *EGFR* mutation and TKI treatment exacerbated immunosuppressive effects of PD-L1-positive sEVs. (**A**) Schematic diagram of the workflow. (**B**) Representative contour plot of the gating strategy of CD8^+^ T cells. (**C**) Representative contour plots of CD8^+^ T cells examined for the expression of Granzyme B. CD8^+^ T cells were treated with sEVs derived from H1264-WT, H1264-L858R+T790M mutation, and H1264-L858R+T790M mutation treated with gefitinib. (**D**) Quantitative analysis of the Granzyme B expression in CD8^+^ T cells. (**E**) Representative contour plots of CD8^+^ T cells examined for the expression of Ki67. CD8^+^ T cells were treated with sEVs derived from H1264-WT, H1264-L858R+T790M mutation, and H1264-L858R+T790M mutation treated with gefitinib. (**F**) Quantitative analysis of the Ki67 expression in CD8^+^ T cells.

Based on the aforementioned result, both activation and inhibition of the EGFR signaling pathway resulted in an increase of the secretion level of small extracellular vesicle PD-L1. However, in these two scenarios, the key molecules regulating the release of PD-L1-positive sEVs were different. It remained unclear whether the PD-L1-positive sEVs possess immunosuppressive functions under these two scenarios. Therefore, to explore the immunosuppressive function of PD-L1-positive sEVs, PBMCs were cultured and activated by CD3/CD28 antibodies in a six-well plate and treated with sEVs (Figure 6A). We collected sEVs from three conditions including H1264 *EGFR*-WT cell line (referred to as WT sEVs), H1264 *EGFR*-L858R+T790M mutation cell line (referred to as L858R+T790M sEVs), and H1264 *EGFR*-L858R+T790M mutation cell line treated with gefitinib (referred to as L858R+T790M-Gef sEVs). PBMCs were treated with indicated sEVs, where each had an equivalent total protein content. CD8 was stained to gate CD8^+^ T cells (Figure 6B). The proliferation and cytotoxicity of CD8^+^ T cells were determined by the expression level of Ki67 and Granzyme B. WT sEVs inhibited function of CD8^+^ T cells, as indicated by decreased expression of Ki67 and Granzyme B (Figure 6C–F). In addition, L858R+T790M and L858R+T790M-Gef sEVs exhibited enhanced immunosuppressive effects, indicating the enhanced immunosuppressive function of PD-L1-positive sEVs secreted by H1264 L858R+T790M cell line and H1264 L858R+T790M cell line treated with gefitinib (Figure 6C–F).

## 4. Discussion

Immunotherapy has achieved remarkable success across a spectrum of cancer types, significantly extending the lives of patients with advanced-stage disease. However, those with *EGFR* mutations display a lower sensitivity to immunotherapy, making it a less recommended course of treatment for this patient subset [18,19,20]. Moreover, these individuals, even after developing resistance to TKI therapies and when other options are exhausted, continue to show resilience to immunotherapy [5,21]. Their response to such treatments is markedly poorer than that of patients without *EGFR* mutations. Consequently, the quest to enable the predominant group of lung cancer patients who harbor *EGFR* mutations to benefit from immunotherapy remains a challenging pursuit. Our present research sheds light on a potential pivotal factor in the resistance to immunotherapy among patients with *EGFR* mutations, sEV-associated PD-L1, offering a fresh angle for examining resistance mechanisms. Our findings indicate that NSCLC cells can produce PD-L1-positive sEVs that inhibit CD8^+^ T cell function, thereby exerting an immunosuppressive effect. *EGFR* mutations promote the release of these PD-L1-positive sEVs from NSCLC cells. Surprisingly, targeting the mutant EGFR pathway with TKIs does not suppress this release; instead, it appears to further increase the secretion of PD-L1-positive sEVs by NSCLC cell lines. This phenomenon might help explain the subpar effectiveness observed in *EGFR*-mutant NSCLC patients, regardless of whether they are administered with direct PD-L1/PD-1 inhibitors or have transitioned to these immunotherapies after developing TKI resistance. Therefore, this study hopes to provide novel insights into the study and enhancement of immunotherapeutic efficacy in *EGFR*-mutant patients.

In recent years, a great deal of research has confirmed that tumor-secreted sEVs play a key role in tumor initiation, immune escape, and treatment resistance [22]. This understanding naturally points to the possibility that hindering the release of sEVs from tumor cells could be a viable strategy in combating cancer [23]. Both our initial investigations and this ongoing research underscore the significance of PD-L1-positive sEVs in promoting immunosuppressive function and therapy resistance. Consequently, blocking the release of these vesicles from tumor cells could potentiate the efficacy of cancer treatments. Unraveling the molecular mechanism behind tumor cell secretion of PD-L1-positive sEVs is essential for developing targeted inhibitors. However, the exact molecular mechanism of formation and release of PD-L1-positive sEVs in tumor cells remains unclear at this time. According to existing literature, some key molecules involved in regulating PD-L1-positive sEVs include HRS, ALIX, and Rab27a [24]. We have therefore verified these molecules one by one. We found that both *EGFR* gene mutation and TKI treatment of tumor cells carrying *EGFR* mutations have little effect on the intracellular level of Rab27a. Thus, Rab27a may not be involved in the process that mediates the secretion of PD-L1-positive sEVs by NSCLC cells. Our investigation reveals that NSCLC cells with *EGFR* mutations express higher levels of HRS (with no significant change in ALIX) and that the expression level of PD-L1 in circulating sEVs from patients is directly tied to HRS levels in their tumor tissues. After TKIs treatment of NSCLC cells (carrying *EGFR* mutations), the increase of PD-L1 levels in sEVs is accompanied by a significant increase in ALIX levels in cells (without significant HRS alterations). Concurrently, patients undergoing TKI therapy experience an increase in PD-L1-positive sEVs in the bloodstream, which correlates with the ALIX expression in their tumor tissues. *HRS* and *ALIX* knockdown experiments have further confirmed the regulatory functions of HRS and ALIX in these contexts. These insights suggest that NSCLC cells adapt and invoke different signaling pathways to sustain the secretion of PD-L1-positive sEVs under varying survival pressures. Moving forward, it is imperative to develop multifaceted inhibitors targeting the secretion of sEV PD-L1, enhancing our ability to impede this process effectively.

Our study does present certain limitations. For example, this study only preliminarily demonstrates the changes in PD-L1 expression levels in sEVs secreted by non-small-cell lung cancer cells under *EGFR* mutation and TKI treatment conditions, as well as the potential underlying molecular mechanisms. Future experiments are needed to prove whether inhibiting the secretion of PD-L1-positive sEVs by *EGFR*-mutant NSCLC tumor cells could synergistically enhance the efficiency of PD-L1/PD-1 inhibitors. However, this work is currently limited by the lack of specific inhibitors for HRS and ALIX. Given the widely confirmed roles of HRS and ALIX in regulating the formation of PD-L1-positive sEVs across various tumors, developing targeted small-molecule inhibitors against these proteins presents an exciting and valuable direction for sEV-based cancer therapeutic research [25].

## 5. Conclusions

In summary, our findings suggest that *EGFR* mutations and TKI treatments are linked to a surge in the release of PD-L1-positive sEVs, which may contribute to immunosuppression and consequently undermine the effectiveness of immunotherapy in patients harboring *EGFR* mutations or those being administered with TKIs. We hope that these insights will pave the way for a novel and effective approach to overcoming resistance to immunotherapy in patients harboring *EGFR* mutations.

## Data Availability

The datasets generated and/or analyzed during the current study are not publicly available due to local legal requirements but are available from the corresponding author upon reasonable request.

## Supplementary Materials

The following supporting information can be downloaded at: https://www.mdpi.com/article/10.3390/biom14070820/s1, Supplementary method; Western Blotting Figures; Figure S1. EGFR mutation promoted expression of cellular PD-L1; Figure S2. EGF stimulation promoted expression of cellular PD-L1; Figure S3. EGF stimulation promoted secretion of sEV PD-L1; Figure S4. EGF stimulation and EGFR mutation promoted expression of HRS; Figure S5. H1975 cell line resisted to gefitinib treatment; Figure S6. TKI treatment promoted expression of ALIX.
