# Supplementary material for: EGFR Mutation and TKI Treatment Promote Secretion of Small Extracellular Vesicle PD-L1 and Contribute to Immunosuppression in NSCLC
Source: Biomolecules. 2024 Jul 9;14(7):820. doi: 10.3390/biom14070820 (PMC11274907; doi:10.3390/biom14070820)
Supplement: Supplementary file 1 [file biomolecules-14-00820-s001.zip › biomolecules-3027689-supplementary.pdf]

Supplementary Materials

For

EGFR mutation and TKI treatment both induced increased small extracellular vesicle PD-L1 contributes to the immunosuppression in NSCLC

Hai-Ming Liu[1†], Zi-Li Yu[1,2 †], Hou-Fu Xia[1,2], Lin-Zhou Zhang[1], Qiu-Yun Fu[1], Yi Wang[3], Hong-Yun Gong[3 *] and Gang Chen[1,2,4,5 *]

1 State Key Laboratory of Oral & Maxillofacial Reconstruction and Regeneration, Key Laboratory of Oral Bio-medicine Ministry of Education, Hubei Key Laboratory of Stomatology, School & Hospital of Stomatology, Wuhan University, Wuhan, China,430079.

2 Department of Oral and Maxillofacial Surgery, School and Hospital of Stomatology, Wuhan University, Wuhan, China,430079.

3 Cancer Center, Renmin Hospital of Wuhan University, Wuhan, China, 430060.

4 TaiKang Center for Life and Medical Sciences, Wuhan University, Wuhan, China, 430071.

5 Frontier Science Center for Immunology and Metabolism, Wuhan University, Wuhan, China, 430071.

* Correspondence: Hong-Yun Gong (drgonghongyun@whu.edu.cn); Gang Chen (geraldchan@whu.edu.cn)

† These authors contributed equally to this work.

Contents

Supplementary methods. 2

Supplementary figure 1. *EGFR* mutation promoted expression of cellular PD-L1 5

Supplementary figure 2. EGF stimulation promoted expression of cellular PD-L1 6

Supplementary figure 3. EGF stimulation promoted secretion of sEV PD-L1 7

Supplementary figure 4. EGF stimulation and *EGFR* mutation promoted expression of HRS 8

Supplementary figure 5. H1975 cell line resisted to gefitinib treatment 9

Supplementary figure 6. TKI treatment promoted expression of ALIX 10

Supplementary Methods

Isolation of extracellular vesicles

For purification of small extracellular vesicles from cell culture supernatants, cells were cultured in media supplemented with 10% extracellular vesicle (EV)-depleted fetal bovine serum (FBS). Bovine EVs were depleted by overnight centrifugation at 120,000 g. Supernatants were collected from 48-72 h cell cultures and small extracellular vesicles were purified by a standard differential centrifugation protocol. In brief, culture supernatants were centrifuged at 3,000 g for 30 min to remove cell debris, dead cells and large small extracellular vesicles (Eppendorf, 5810R). Then culture supernatants were centrifuged at 120,000 g for 1.5 h at 4 °C (Beckman Coulter, Optima XPN-100). The pelleted extracellular vesicles were suspended in PBS.

For purification of circulating extracellular vesicles by differential centrifugation, peripheral blood samples were subjected to centrifugation at 1550 g for a duration of 30 min to separate the cell-free plasma (Eppendorf, 5810R). Subsequently, 1 mL of the isolated cell-free plasma underwent centrifugation at 3,000 g for a duration of 30 min (Eppendorf, 5810R) in order to remove large small extracellular vesicles. At last, the plasma was centrifuged at 120,000 g for 1.5 h at 4 °C (Beckman Coulter, Optima MAX-XP). The pelleted extracellular vesicles were suspended in PBS

Quantification of the sEV secretion capacity of cell lines

The sEV secretion capacity of cell lines were measured by quantity of sEVs released per cell, calculated as the total count of sEVs divided by the cell count. The protein content of sEVs was calculated as the total protein amount divided by the number of sEVs.

Transmission electron microscopy (TEM)

Fix the sEVs samples in a glutaraldehyde-containing buffer to preserve their structure and stain the sEVs samples with uranyl acetate to enhance contrast. Drop the fixed and stained sEVs samples onto copper grids and allow them to air dry. The prepared samples were detected using transmission electron microscope (Hitachi).

Plasmids construction

Two short hairpin RNAs (shRNA) targeting human *HRS* 5′-GCACGTCTTTCCAGAATTCAA-3′, 5′-GCATGAAGAGTAACCACAGC-3′ and one shRNA targeting human *ALIX* 5′-GCCGCUGGUGAAGUUCAUCTT-3′ were cloned into the pLKO.1-Puro vector. The full length of *EGFR*-WT, *EGFR*-L858R and *EGFR*-L858R+T790M cDNA were cloned into the pLenti-CMV-EGFP-3FLAG-PGK-blasticidin. The constructed plasmids were transfected into HEK293T cells with Lipofectamine 3000 (Invitrogen) according to the manufacturer's

instructions. Viral supernatants were harvested at 48 h after transfection and centrifuged at 3,000 g for 30 min for infection. Virally infected cells were selected with puromycin or blasticidin to achieve high efficiency of genetic interference.

Immunofluorescence staining

Immunofluorescence staining was conducted on cells that had been fixed. After blocking and permeabilization, the fixed cells were incubated with primary antibodies overnight at 4 °C. After washing with PBS, the fixed cells were then incubated with fluorophore-conjugated secondary antibodies for 1 h. DAPI was used to stain the nuclei. The images of the samples were obtained by a Leica confocal microscope at 100 × magnification.

Real-Time Quantitative Polymerase Chain Reaction (RT-qPCR) assay

Total cell RNAs were isolated and reverse transcribed into cDNA. Polymerase chain reaction (PCR) was performed using 7900HT Real-time PCR System (Applied Biosystems). Detailed steps and the primer sequences are provided in the supplementary material. The primer sequences used for RT-qPCR were listed as follows: *HRS*: 5′-TGATCTGATGCATGT-3′ *ALIX*: 5′-TGATCTGATGGTCTA-3′ *Rab27a*: 5′-GCTTTGGGAGACTCTGGTGTA-3′, *β-actin*: 5′-TGCTGTCTCGTATG-3′. *β-actin* was selected as the internal control for each experiment. The mRNA expression levels were assessed using evaluated threshold cycle (CT) values. Consistent with prior research reported, the CT values were standardized by the *β-actin* expression level. The 2−ΔΔCT method was employed to calculate the relative quantity of mRNA.

Enzyme-linked immunosorbent assay (ELISA)

96-well ELISA plates were coated with anti-PD-L1 overnight at 4 °C. The solution from each well was then removed, and 200 μL of wash buffer was added per well, followed by washing with PBS four times for 5 minutes each. Next, 200 μL of blocking buffer was added to each well, incubated for 1 hour at room temperature (RT), and washed four times for 5 minutes each. Then, sEV samples and standard proteins were added and incubated for 1 hour at RT. Biotinylated anti-PD-L1, diluted in 100 μL blocking buffer, was added to each well and incubated for 1 hour at RT. After washing with PBS, 100 μL of enzyme-conjugated secondary antibodies were added to the plate and incubated for 1 hour at RT. The wells were then washed 5 times for 5 minutes each, and 100 μL of horseradish peroxidase (HRP) substrate solution (1 mg/mL TMB) was added. The plate was incubated until it turned blue, and 100 μL of stop solution was added. Finally, the plate was read at 450 nm absorbance using a PowerWave XS2 (BioTek) for quantification.

Immunohistochemistry

The IHC staining was performed using anti-p-EGFR (1:100, #2234S, Cell Signaling Technology), anti-HRS (1:200, #10390-1-AP, Proteintech), anti-ALIX (1:200, # ab117600, Abcam) and anti-CD8

(1:400, #85336S, Cell Signaling Technology). Membrane, nuclear, or pixel immunohistochemical staining was scored based on the staining intensity and the percentage of different positive cells. The score was obtained by applying the following formula, Histoscore = [(percentage of weakly positive staining) × 1 + (percentage of moderately positive staining) × 2 + (percentage of strongly positive staining) × 3]. The density of CD8+ infiltrating T cells was determined as the mean number of cells/mm2 from 3 randomized fields.

Western blotting analysis

An equivalent amount of protein was separated using sodium dodecyl sulfate–polyacrylamide gel and transferred via electrophoresis onto a polyvinylidene fluoride membranes. After blocking, the membranes were incubated with primary antibodies overnight at 4 °C. EGFR (1:1000, #4267S, Cell Signaling Technology), Calnexin (1:1000, #2679S, Cell Signaling Technology), HRS (1:1000, #15087S, Cell Signaling Technology), ALIX (1:1000, #634502, Biolegend), Rab27a (1:1000, #69295S, Cell Signaling Technology), p-EGFR (1:1000, #2234S, Cell Signaling Technology), CD63 (1:1000, ab134045, Abcam), PD-L1 (1:1000, #13684S, Cell Signaling Technology) and β-actin (1:1500, #AC026, Abclonal), GAPDH (1:1500, #AC002, Abclonal)

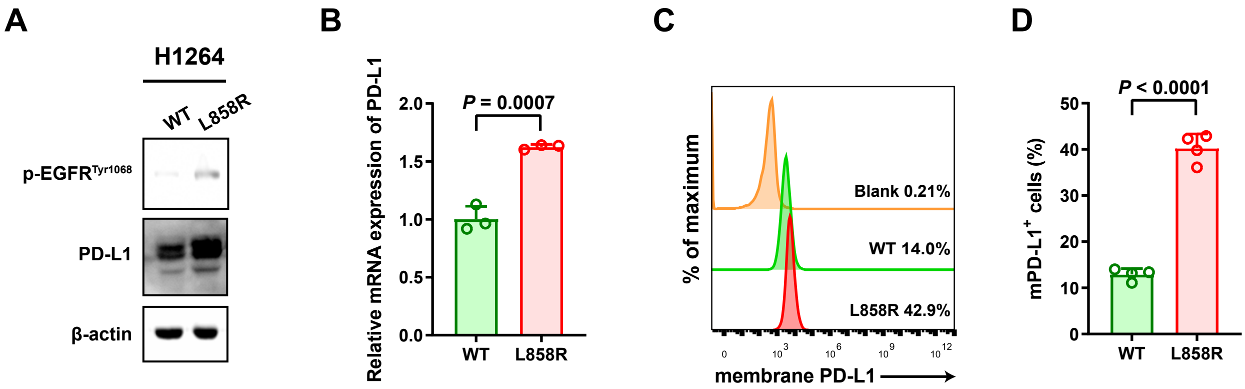

Figure S1. ***EGFR*** **mutation promoted expression of cellular PD-L1.**

A.Western blotting analysis of PD-L1 in WT and L858R mutation H1264 cell lines.

B. RT-PCR of PD-L1 in WT and L858R mutation H1264 cell lines.

C. Representative histograms of flowcytometry analysis for the membrane PD-L1 of WT, L858R mutation H1264 cell lines.

D. Quantification of the percentage of membrane PD-L1. Statistical analyses were performed using a two-tailed unpaired t test.

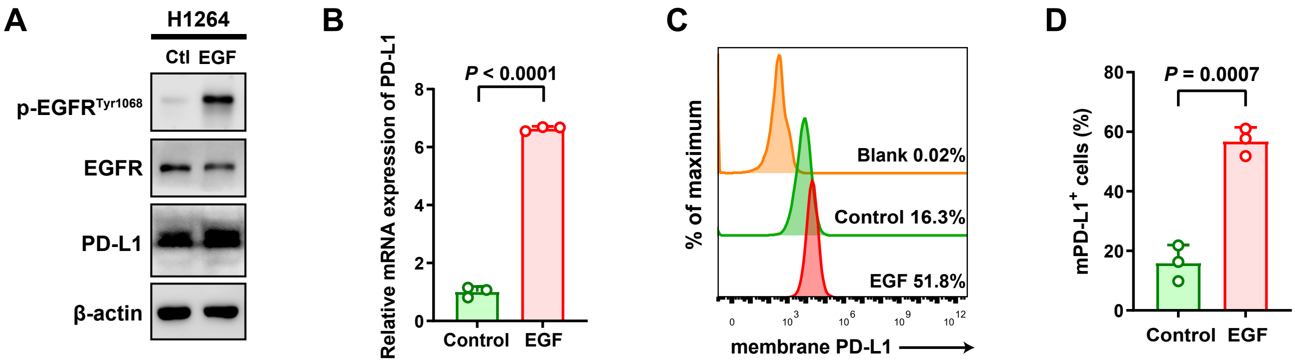

Figure S2. EGF stimulation promoted expression of cellular PD-L1.
A. Western blotting analysis of PD-L1 in H1264 cell lines stimulated with EGF.
B. RT-PCR of PD-L1 in H1264 cell lines stimulated with EGF.
C. Representative histograms of flowcytometry analysis for the membrane PD-L1 of H1264 cell lines stimulated with EGF.
D. Quantification of the percentage of membrane PD-L1. Statistical analyses were performed using a two-tailed unpaired t test.

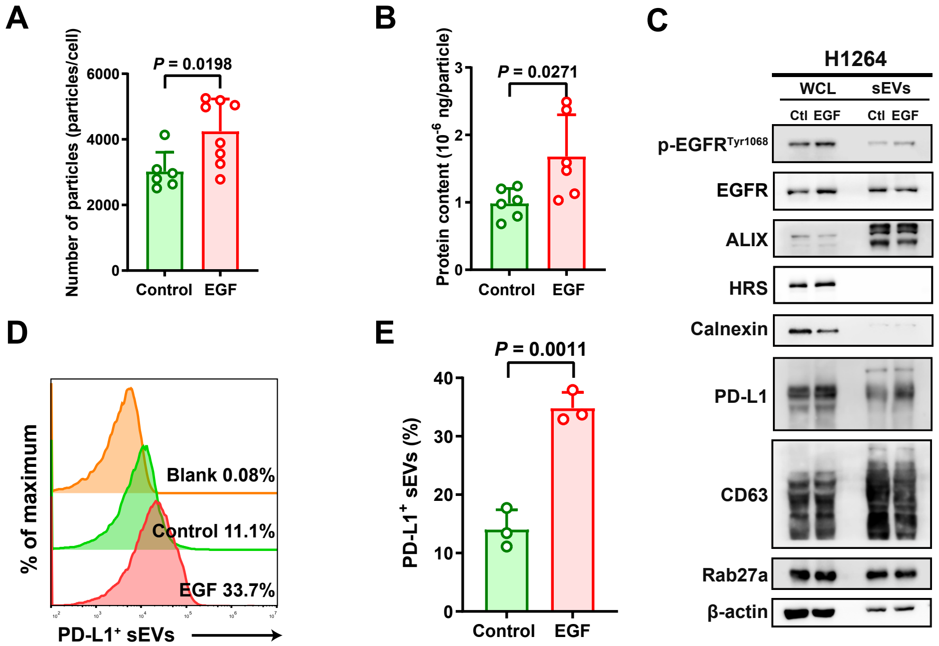

Figure S3. EGF stimulation promoted secretion of sEV PD-L1.

A. Number of particles detected in the cultural supernatant of H1264 cell lines stimulated with EGF.
B. Protein content of particles detected in the cultural supernatant of H1264 cell lines stimulated with EGF.
C. Western blotting analysis of HRS and PD-L1 in EGF stimulated H1264 cell lines and the derived sEVs. The same protein amount of whole cell lysate and the same number of sEVs was loaded in each lane.
D. Representative histograms of high-resolution flowcytometry for the sEV PD-L1 from the supernatant of H1264 cell lines stimulated with EGF.
E. Quantification of the percentage of sEV PD-L1. Statistical analyses were performed using a two-tailed unpaired t test.

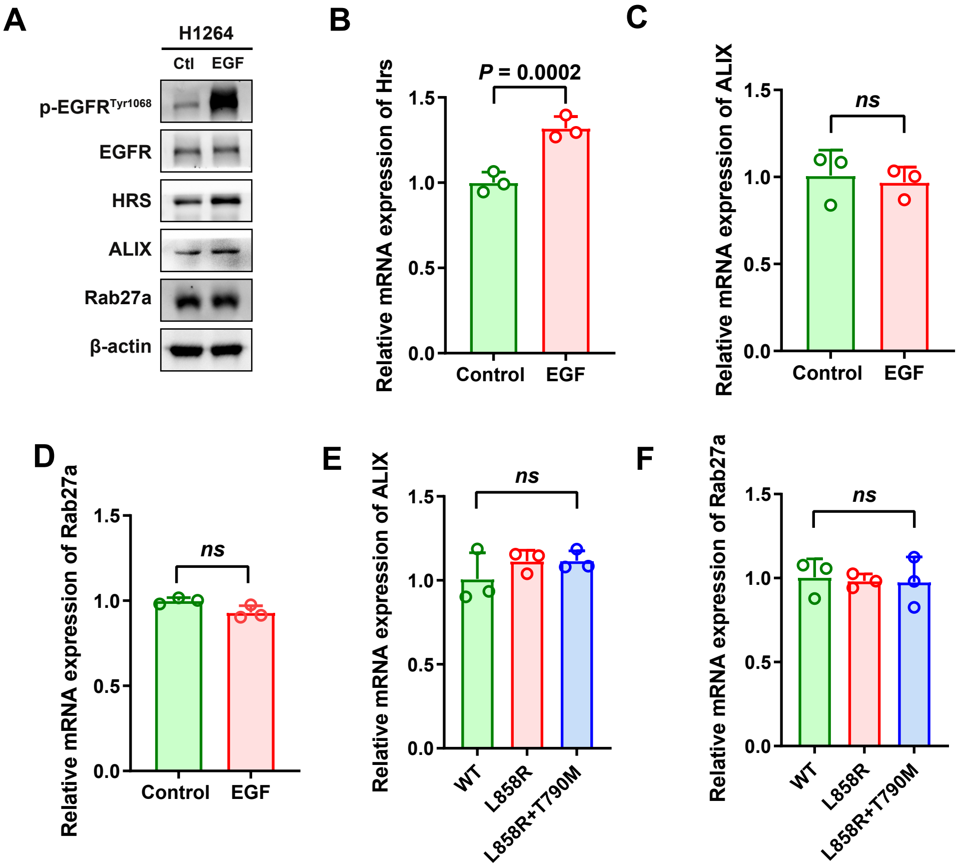

Figure S4. EGF stimulation and *EGFR* mutation promoted expression of HRS.
A. Western blotting analysis of HRS, ALIX and Rab27a in H1264 cell lines stimulated with EGF.
B. RT-PCR of HRS in H1264 cell lines stimulated with EGF.
C. RT-PCR of ALIX in H1264 cell lines stimulated with EGF.
D. RT-PCR of Rab27a in H1264 cell lines stimulated with EGF.
E. RT-PCR of ALIX in WT, L858R and L858R+T790M mutation H1264 cell lines.
F. RT-PCR of Rab27a in WT, L858R and L858R+T790M mutation H1264 cell lines.

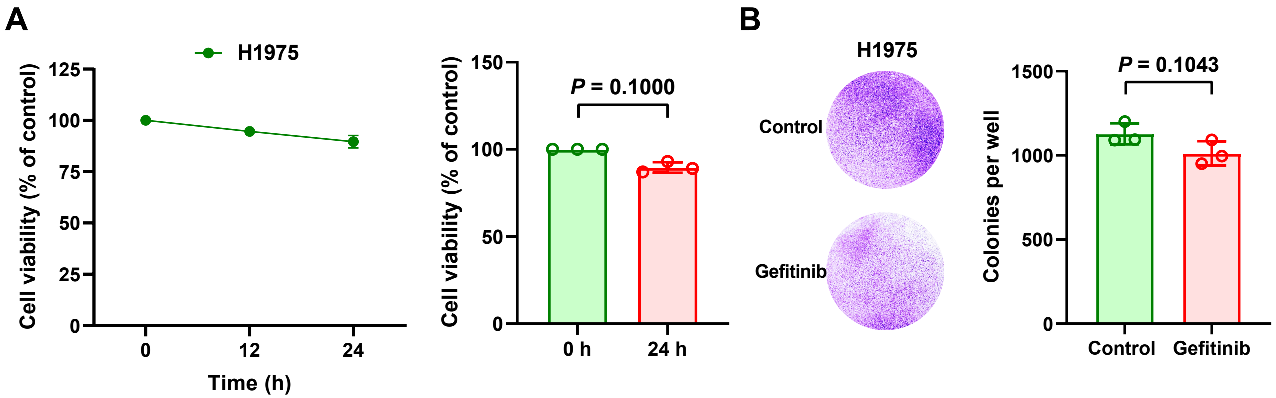

Figure S5. H1975 cell line resisted to gefitinib.
A. CCK8 assay of H1975 cell lines treated with gefitinib.
B. Colony formation assay of H1975 cell lines treated with gefitinib.

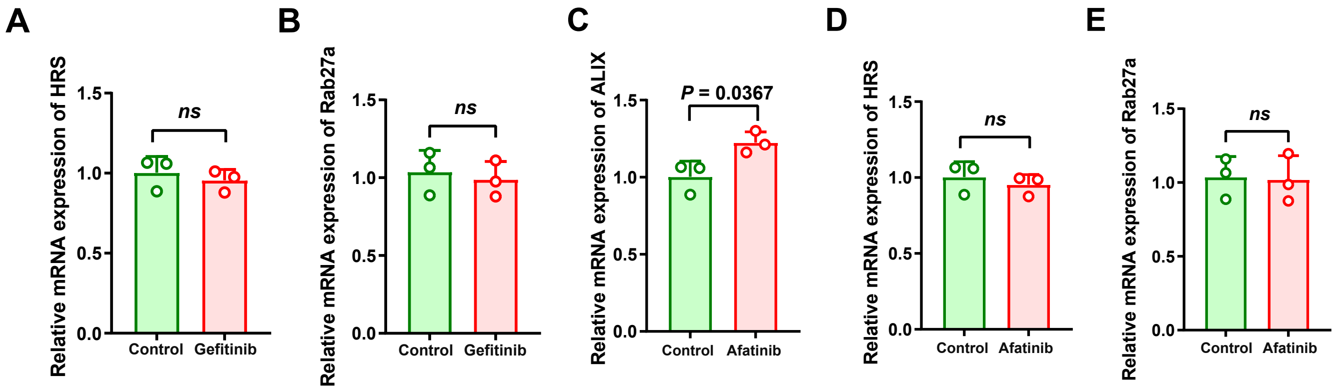

Figure S6. TKI treatment promoted expression of ALIX.
A. RT-PCR of HRS in H1975 cell lines treated with gefitinib.
B. RT-PCR of Rab27a in H1975 cell lines treated with gefitinib.
C. RT-PCR of ALIX in H1975 cell lines treated with afatinib.
D. RT-PCR of HRS in H1975 cell lines treated with afatinib.
E. RT-PCR of Rab27a in H1975 cell lines treated with afatinib.
